# Prognostic Value of Fibrinogen-to-Albumin Ratio and Neutrophil-to-Lymphocyte Ratio in Patients on Peritoneal Dialysis

**DOI:** 10.3390/life15111728

**Published:** 2025-11-09

**Authors:** Selena Gajić, Ana Bontić, Aleksandar Sič, Vidna Karadžić-Ristanović, Milorad Stojadinović, Kristina Filić, Jelena Pavlović, Jovana Gavrilović, Kristina Petrović, Sanja Stanković, Nikola Trnić, Filip Simović, Pavle Popović, Svetlana Jovičić-Pavlović, Aleksandra Kezić, Marko Baralić

**Affiliations:** 1 Clinic of Nephrology, University Clinical Center of Serbia, Pasterova 2, 11000 Belgrade, Serbia; 2 Faculty of Medicine, University of Belgrade, Dr Subotića Starijeg 8, 11000 Belgrade, Serbia; 3 Center for Medical Biochemistry, University Clinical Center of Serbia, 11000 Belgrade, Serbia; 4 Faculty of Medical Sciences, University of Kragujevac, 34000 Kragujevac, Serbia; 5 Clinic for Emergency Surgery, Emergency Center, University Clinical Center of Serbia, Pasterova 2, 11000 Belgrade, Serbia

**Keywords:** peritoneal dialysis, mortality, chronic inflammation, inflammatory markers, neutrophil-to-lymphocyte ratio, fibrinogen-to-albumin ratio

## Abstract

Background and Objectives: Chronic inflammation (CIn) is common among peritoneal dialysis (PD) patients and contributes to adverse outcomes. However, the prognostic value of the neutrophil-to-lymphocyte ratio (NLR) and fibrinogen-to-albumin ratio (FAR) in PD remains uncertain. Methodology: In this prospective cohort study, 65 PD patients were followed for 18 months. Baseline demographic, clinical and laboratory data were collected and inflammatory indices were calculated. The composite outcome was all-cause mortality or transfer to hemodialysis (HD). Logistic regression analyses were used to identify independent predictors of outcomes. Results: Over the 18-month follow-up, 23 patients (35.4%) died and 13 (20.0%) transferred to HD. Patients with adverse outcomes had higher baseline FAR, C-reactive protein (CRP) and serum glucose (Glc) levels and lower triglycerides (TG). In multivariate analysis, higher FAR (OR 5.28, 95% CI 1.16–24.12), CRP (OR 1.28, 95% CI 1.02–1.62) and PTH (OR 1.01, 95% CI 1.00–1.01) were independently associated with adverse outcomes, while NLR showed marginal significance. In the mortality-only model, FAR (OR 3.99, 95% CI 1.17–13.61) and PTH remained significant predictors. Conclusions: FAR demonstrated a significant prognostic association with mortality and composite adverse outcomes in PD patients, whereas NLR had limited predictive value. Albumin-based inflammatory indices such as FAR may complement established markers for risk stratification. Larger multicenter studies are warranted to validate these findings.

## 1. Introduction

Chronic inflammation (CIn), defined as the persistence of elevated inflammatory markers for more than three months, is a hallmark of end-stage kidney disease (ESKD) [1]. It is commonly manifested through hyperfibrinogenemia, hypoalbuminemia, leukocytosis with a shift in leukocyte lineages and elevated C-reactive protein (CRP) levels, accompanied by metabolic alterations such as changes in body mass index (BMI) and hemoglobin concentration [1,2,3]. In patients with ESKD, CIn has been strongly associated with increased cardiovascular disease (CVD) risk and poor survival outcomes [2].

Although numerous inflammatory biomarkers have been investigated in hemodialysis (HD) patients, many are not routinely measured in clinical practice due to cost or complexity [1]. In recent years, attention has shifted toward ratios derived from standard laboratory tests, which are inexpensive, reproducible and widely available. The neutrophil-to-lymphocyte ratio (NLR), calculated as the neutrophil count divided by the lymphocyte count (Ly) [1] and the fibrinogen-to-albumin ratio (FAR), defined as plasma fibrinogen (Fib) divided by serum albumin (Alb) [2], have emerged as promising indicators of systemic inflammation [4].

Several studies have reported that an elevated NLR is associated with increased inflammation and mortality among HD patients [3,5], while FAR has demonstrated prognostic value across multiple clinical settings, including chronic kidney disease (CKD) [4]. However, evidence regarding the prognostic significance of these markers in peritoneal dialysis (PD) remains limited and inconsistent [4,5,6].

While several prior studies [2,4,7,8] have demonstrated the prognostic relevance of FAR in patients with CKD and those undergoing HD, evidence in PD population is still limited. The present study builds on these findings by using a prospective design focused exclusively on PD patients and by evaluating both mortality and transfer to HD as a composite outcome. This approach captures broader technique-failure events relevant to PD viability and enables a direct comparison between FAR and NLR—two readily available inflammatory indices with distinct biological underpinnings. In doing so, the study extends prior research on FAR and NLR to a PD-specific context, providing novel insight into their relative prognostic value in this population.

## 2. Materials and Methods

### 2.1. Ethics Statements and Study Population

A total of 65 patients undergoing PD at the Clinic of Nephrology, University Clinical Center of Serbia (UCCS), were enrolled in December 2019 and prospectively followed for 18 months. The study protocol was approved by the Ethics Committees of UCCS (no. 890/8, 21 December 2018).

All participants provided written informed consent prior to inclusion and the study was conducted in accordance with the principles of the Declaration of Helsinki. Patients with peritonitis or clinical and/or laboratory signs of PD catheter exit-site infection within three months before enrollment were excluded.

### 2.2. Data Collection and Laboratory Analyses

Baseline demographic, clinical and laboratory data were recorded at enrollment. Collected variables included sex, age, PD duration, PD modality, use of icodextrin solution, daily ultrafiltration, daily urine output, cause of ESKD and comorbidities such as hypertension (HTN), diabetes mellitus (DM), ischemic heart disease (IHD), cerebrovascular insult (CVI), peripheral vascular disease (PVD), dementia, chronic obstructive pulmonary disease (COPD), peptic ulcer disease, liver disease, connective tissue disease, leukemia/lymphoma and cancer or acquired immunodeficiency syndrome (AIDS). 

Fasting blood samples were obtained in the morning. Hematological parameters were analyzed using the Beckman Coulter^®^ HmX Hematology Analyzer (Beckman Coulter, Inc., Brea, CA, USA), while biochemical parameters were measured with the Architect ci8200 system (Abbott Diagnostics, Wiesbaden, Germany). Laboratory data included CRP, serum glucose (sGlc), serum urea (sUr), serum creatinine (sCr), total cholesterol (Chol), triglycerides (TG) and parathyroid hormone (PTH). The NLR was calculated as the ratio of neutrophil to Ly counts. The FAR was calculated as plasma Fib (g/L) divided by serum Alb (g/L), ensuring both parameters were expressed in the same units prior to calculation. PD treatment adequacy was assessed using Kt/V and total weekly creatinine clearance (CCr, L/week), calculated with PD Adequest software 2.0 (Baxter Healthcare Ltd., Deerfield, IL, USA). Peritoneal transport characteristics were evaluated using the Peritoneal Equilibration Test (PET), performed according to the recommendations of Cnossen et al. [9].

### 2.3. Outcome Measures

Patients were followed until death, transfer to HD, kidney transplantation, referral to another center, or study completion. The primary composite outcome was defined as all-cause mortality or transfer to HD.

The composite endpoint was selected to capture major adverse outcomes to PD technique failure. Both death and transfer to HD represent clinically meaningful endpoints that reflect the loss of PD viability. The use of these composite outcomes is consistent with the concept of Major Adverse Renal Events (MARE), commonly used in PD outcome studies. 

### 2.4. Statistical Analysis

Data were coded, tabulated and varified for accuracy prior to analysis. Continuous variables were expressed as mean ± standard deviation (SD) or median [interquartile range], depending on distribution, while categorical variables were presented as absolute numbers (*n*) and percentages (%). Normality of continuous variables was assessed before applying parametric or nonparametric tests. Between-group comparisons were conducted using the independent samples t-test or Mann–Whitney U test for continuous variables and chi-square or Fisher’s exact test for categorical variables.

All assumptions for logistic regression were evaluated before model construction. Due to collinearity between PD treatment adequacy (Kt/V) and the Charlson Comorbidity Index (CCI), only Kt/V was retained in the final models. Variables violating linearity assumptions (CCI, period on PD and FAR) were either log-transformed or standardized as z-scores to improve model fit. Outliers were identified by standardized residuals >2.5 and excluded prior to analysis. Binary logistic regression was then performed to identify predictors of the composite outcome and 18-month mortality.

All statistical analyses were performed using IBM SPSS Statistics, version 18.0 (SPSS Inc., Chicago, IL, USA). A two-tailed *p*-value < 0.05 was considered statistically significant.

Model performance was internally validated by assessing both discrimination and calibration. Discrimination was evaluated using receiver operating characteristic (ROC) curve analysis, with the area under the curve (AUC) used to quantify overall predictive accuracy. Calibration was examined with the Hosmer–Lemeshow goodness-of-fit test. ROC curves for both logistic regression models (composite outcome and 18-month mortality) were plotted to visually illustrate discriminative power.

## 3. Results

A total of 65 patients were included, of whom 30 (46.2%) were female. The median age was 63 [16] years. The leading causes of ESKD were hypertensive nephrosclerosis (38.4%), diabetic nephropathy (27.7%), chronic glomerulonephritis (20.0%), tubulointerstitial nephritis (3.1%), autosomal dominant polycystic kidney disease (1.5%) and other causes (9.2%). The most frequent comorbidities were IHD (18.5%), PVD (15.4%) and CVI (7.7%). HTN and DM were analyzed as primary causes of ESKD and, therefore, not additionally classified as comorbidities. Most patients (*n* = 59, 90.8%) were on continuous ambulatory peritoneal dialysis (CAPD), while 6 (9.2%) were on automated peritoneal dialysis (APD). Median duration of PD treatment was 22 [42] months.

During the 18 months follow-up, 23 patients (35.4%) died and 13 (20.0%) were transferred to HD. No patients underwent kidney transplantation or were referred to another center. Baseline demographic and clinical characteristics are summarized in Table 1 and baseline laboratory data are presented in Table 2.

When patients were stratified according to outcomes, 29 remained on PD and constituted the technique survival group, while 36 experienced either death or transfer to HD, forming the composite outcome group. There were no significant differences between the two groups in baseline demographic characteristics, dialysis adequacy parameters, or overall comorbidity burden, except that a significantly higher proportion of patients in the composite outcome group had cardiomyopathy (CMP). However, a significant difference was observed in the primary cause of ESKD (*p* = 0.036), with chronic GN being more prevalent in PD technique survival and diabetic nephropathy more common in the composite outcome group (Table 3).

Patients in the composite outcome group exhibited higher baseline levels of FAR (*p* = 0.07), CRP (*p* < 0.01) and sGlc (*p* = 0.022), along with lower TG concentrations (*p* = 0.040), compared with the PD technique survival group. No significant between-group differences were observed in sUr, sCr, Chol, PTH, or NLR (Table 4).

**Figure 1:**
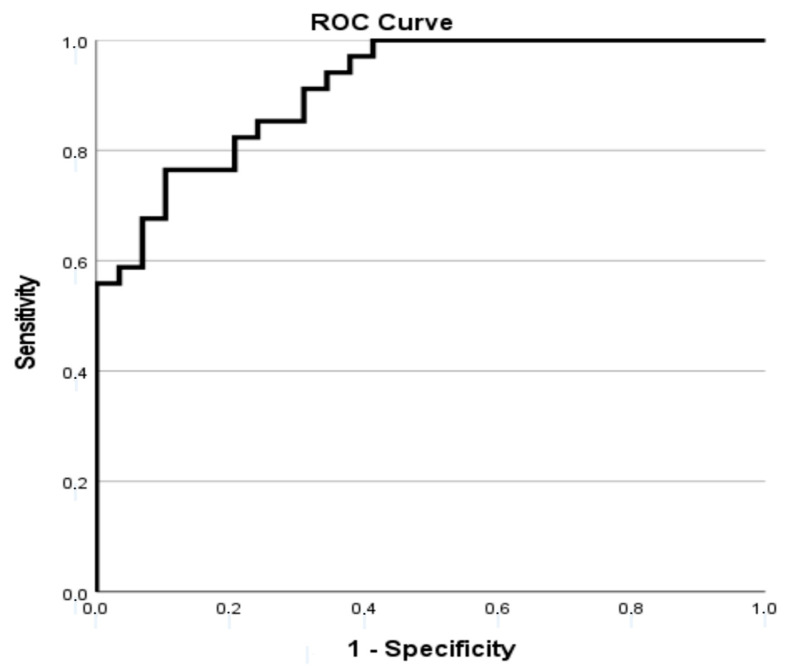
ROC curve for the logistic regression model predicting 18-month composite outcome (death or transfer to hemodialysis) among peritoneal dialysis patients (AUC = 0.913, *p* < 0.001).

After exclusion of two extreme outliers, a logistic regression model was constructed to identify predictors of the 18-month composite outcome (death or transfer to HD). The final model satisfied all analytical assumptions, including the absence of multicollinearity and adequate linearity of continuous predictors. Internal validation demonstrated excellent discriminative ability (AUC = 0.913) and good calibration (Hosmer–Lemeshow test: χ^2^ = 1.366, *p* = 0.995). The ROC curve for this model is presented in Figure 1, demonstrating excellent discrimination between patients with and without adverse outcomes (AUC = 0.913). The model explained 66% of the variance (Nagelkerke R^2^ = 0.658) and achieved an overall classification accuracy of 79.4%. Variables included in the model were: sex, daily urine volume, Kt/V, NLR, TG, PTH, CRP, dialysis modality (CAPD vs. APD), log-transformed CCI, log-transformed period on PD, and standardized FAR.

Logistic regression identified APD use (OR = 76.1, *p* = 0.030), higher baseline PTH (OR = 1.007, *p* = 0.006), CRP (OR = 1.284, *p* = 0.032) and standardized FAR (OR = 5.285, *p* = 0.032) as independent predictors of the composite outcome. NLR (*p* = 0.083) and TG (*p* = 0.063) demonstrated marginal associations. Significant (*p* < 0.05) and marginally significant (*p* < 0.1) predictors are summarized in Table 5.

**Figure 2:**
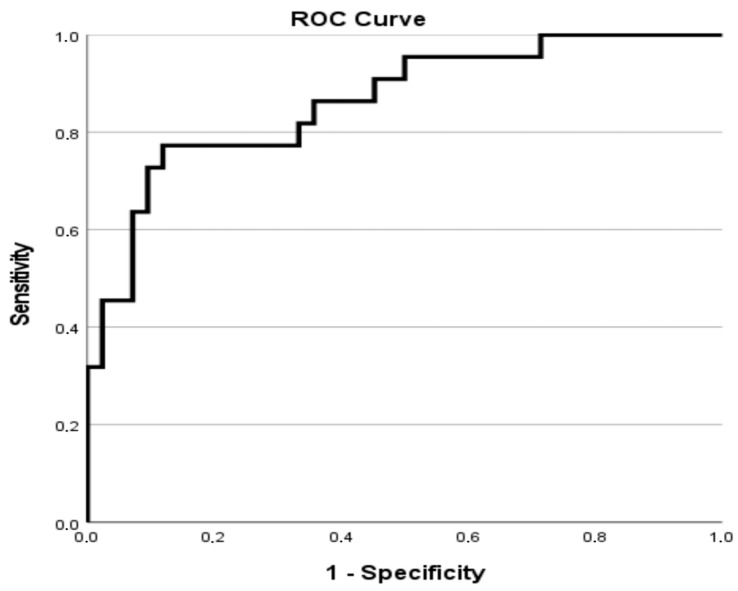
ROC curve for the logistic regression model predicting 18-month mortality among peritoneal dialysis patients (AUC = 0.863, *p* < 0.001).

After excluding one extreme outlier, a second logistic regression model was constructed to identify predictors of 18-month mortality. As in the previous model, all assumptions were satisfied. The model demonstrated good calibration (Hosmer–Lemeshow test, χ^2^ 5798, *p* = 0.670) and strong discriminative ability (AUC = 0.863). The corresponding ROC curve is presented in Figure 2, confirming good discrimination for mortality prediction (AUC = 0.863). It explained 49% of the variance (Nagelkerke R^2^ = 0.489) and achieved an overall classification accuracy of 82.8%. The same set of variables was included as in the composite outcome model.

In the mortality-only model, higher baseline FAR (OR = 3.990, *p* = 0.027) and PTH (OR = 1.005, *p* = 0.015) emerged as independent predictors of mortality, whereas TG showed a borderline association (*p* = 0.095). CRP and PD modality were not significant predictors in this model. Significant (*p* < 0.05) and marginally significant (*p* < 0.1) predictors are presented in Table 6.

## 4. Discussion

Unlike most previous studies based on retrospective or single-endpoint analyses, this study prospectively evaluated the prognostic relevance of FAR and NLR in a PD cohort, incorporating both mortality and technique failure (transfer to HD) as outcomes.

ESKD is the final stage of CKD and is most commonly caused by DM, HTN or GN [10,11,12]. In our cohort, DM and HTN accounted for nearly two-thirds of cases, consistent with global epidemiology. Despite advances in dialysis technology, patients with ESKD continue to experience markedly increased mortality, predominantly due to CVD [13]. During the 18-month follow-up, more than one-third of our patients died, underscoring the persistently poor prognosis in this population. It is noteworthy that this study was conducted during the COVID-19 pandemic, which may have influenced mortality patterns, although reliable cause-of-death data were unavailable. Furthermore, analysis of primary causes of ESKD and comorbidities revealed that a significantly higher proportion of patients in the composite outcome group (death or transfer to HD) had diabetes nephropathy and CMP. This finding aligns with previous reports demonstrating poorer prognosis among patients with DM compared to those without, primarily due to increased cardiovascular morbidity [14]. Although the mortality rate in our study was high, it was comparable to that reported for PD populations in other countries during the COVID-19 pandemic [15].

CIn is increasingly recognized as a nontraditional risk factor for CVD in dialysis patients. It contributes to endothelial dysfunction, a proatherogenic milieu and protein-energy wasting, all of which accelerate morbidity and mortality [14,15]. In PD, inflammation arises from both dialysis-related and systemic factors, including catheter biofilm formation, dialysate bioincompatibility and chronic Glc exposure [16]. Although patients with active infection at baseline were excluded, inflammatory markers still correlated with adverse outcomes, underscoring the ongoing influence of subclinical inflammation in PD. In our cohort, hypertensive nephrosclerosis accounted for 38.4% of ESKD cases. Although primarily a hemodynamic and vascular disorder, hypertensive nephrosclerosis is increasingly recognized as being accompanied by low-grade CIn. Microvascular injury, oxidative stress and endothelial dysfunction promote cytokine release and hepatic synthesis of acute-phase proteins (Fib and CRP), thereby elevating inflammatory indices even in non-glomerular CKD etiologies [17,18].

The biological rationale for the prognostic relevance of FAR and NLR is well established. Both indices reflect systemic inflammation and nutritional status, two key determinants of clinical outcomes in dialysis. Fib is a positive acute-phase reactant associated with endothelial injury, thrombosis and the malnutrition–inflammation–atherosclerosis (MIA) syndrome [19,20,21]. Alb, conversely, declines in CIn and malnutrition and its reduction independently predicts mortality in PD and HD populations [22]. The FAR integrates these opposing biological responses, increased Fib (inflammation and thrombosis) and decreased Alb (malnutrition and protein-energy wasting), making it a more comprehensive indicator than either component alone. This dual nature allows FAR to better capture CIn–catabolic state that underlies adverse outcomes in dialysis patients [23,24]. Similarly, NLR reflects the balance between innate and adaptive immune response and correlates with oxidative stress and uremic inflammation [25]. However, both indices are nonspecific and their levels may fluctuate due to acute infection, cardiovascular events, or transient inflammatory states, which limits their standalone prognostic reliability.

Elevated Fib may promote a pro-thrombotic milieu through increased plasma viscosity, platelet activation and endothelial dysfunction, potentially linking FAR to the MIA process and accelerated atherosclerosis. Concurrent hypoalbuminemia further amplifies these effects by reducing antioxidant capacity and impairing uremic toxin binding [26]. Thus, the observed association between high FAR and mortality in PD may be mediated by endothelial damage, chronic microinflammation and impaired nutritional reserves.

The associations observed between FAR, NLR and adverse outcomes in our cohort are consistent with findings from recent meta-analyses. Although not specific to dialysis, Chen et al. [27] demonstrated that elevated NLR independently predicts both all-cause and cardiovascular mortality in patients with chronic inflammatory diseases, such as COPD, supporting the broader applicability of NLR as a systemic inflammatory marker relevant to CKD and dialysis populations. Similarly, Zou et al. [2] confirmed that higher FAR levels are associated with increased all-cause and cardiovascular mortality in both CKD and PD cohorts. More recent studies have reinforced these observations, although effect sizes vary depending on study design, population and follow-up duration [28,29]. Collectively, these findings support the prognostic relevance of inflammatory ratios, although their predictive strength appears to vary across studies.

Other Alb-based indices have also been explored in dialysis populations. The C-reactive protein-to-albumin ratio (CAR) has been showen to independently predict mortality in PD [30] and higher CAR and ferritin levels have been observed in non-survivors compared to survivors [31]. Similarly, the ferritin-to-albumin ratio has been associated with adverse outcomes [32]. Collectively, these findings suggest that multiple Alb-based ratios, including FAR, CAR, ferritin-to-albumin and albumin/total cholesterol ratio, may complement existing risk models and serve as simple, accessible adjunctive tools for risk stratification in PD patients.

From a clinical perspective, FAR represents a simple, inexpensive and easily repeatable biomarker that could be integrated into routine PD follow-up to identify high-risk individuals who may benefit from closer monitoring or early intervention. Periodic FAR monitoring could complement established markers such as CRP, interleukin 6 (IL-6) and Alb trends within existing nutritional or inflammatory scoring systems [28,29,33]. However, potential multicollinearity among CRP, FAR and PTH should be considered, as these parameters are interrelated through inflammatory and nutritional pathways.

Overall, our findings suggest that FAR and to a lesser extent NLR, provide valuable insight into the inflammatory–nutritional burden of PD patients. These indices may serve as adjunctive, low-cost markers for identifying individuals at increased risk of mortality or technique failure.

However, given the limited sample size and lack of external validation, their clinical application should remain exploratory until confirmed by larger, multicenter, time-to-event studies designed to evaluate their incremental prognostic value. Future studies should also aim to standardize cutoff values and determine whether longitudinal monitoring of FAR can improve long-term outcomes in PD populations.

### Limitations

This study has several important limitations. It was single-centered, included a modest number of patients and assessed inflammatory and nutritional biomarkers only at baseline. The relatively small sample size and limited number of events (36 composite outcomes and 23 deaths) may have contributed to model instability, wide confidence intervals, and potential overfitting in the logistic regression analyses. Future multicenter studies with larger cohorts are warranted to validate these findings and should consider using penalized regression methods (e.g., LASSO or ridge regression) or time-to-event models, such as Cox proportional hazards analysis, to enhance model stability and interpretability when event counts are limited.

Furthermore, external validation was not performed and some relevant confounders (residual kidney function, statin therapy, infection episodes) were unavailable for adjustment. The cohort also included a small number of patients with malignancy, hematologic disease or AIDS. Although these conditions can independently influence inflammatory and nutritional indices (fibrinogen, albumin and leukocyte parameters), they were not excluded due to the limited sample size, which may have introduced additional variability into the results. The inability to analyze cause-specific mortality due to the impact of the COVID-19 pandemic further restricts interpretation.

Nevertheless, the prospective design and systematic follow-up enhance the internal validity of the findings, which should be confirmed in larger, multicenter studies with extended follow-up and standardized biomarker assessment.

## 5. Conclusions

In this prospective study of peritoneal dialysis patients, both the FAR and were independently associated with adverse outcomes, including mortality and transfer to hemodialysis. FAR showed slightly stronger discriminative performance than NLR, likely reflecting its combined inflammatory and nutritional components. These results support the potential role of simple laboratory indices in identifying patients at increased risk of PD technique failure or death. However, due to the limited sample size, low event count and single-center design, the findings should be interpreted cautiously. FAR and NLR may serve as accessible, adjunctive indicators for risk stratification in PD, warranting further validation in larger, multicenter cohorts before clinical implementation.

## Figures and Tables

**Table 1:** Baseline demographic and clinical characteristics of the study population (*N* = 65).

Variables	Total
Age, years	63 [16]
Female, *n* (%)	30 (46.2)
Primary cause of ESKD, *n* (%)	
Hypertensive nephrosclerosis	25 (38.4)
Diabetic nephropathy	18 (27.7)
Chronic GN	13 (20.0)
TIN	2 (3.1)
ADPKD	1 (1.5)
Other	6 (9.2)
Comorbidities, *n* (%)	
CMP	12 (18.5)
AMI	12 (18.5)
CVI	5 (7.7)
PVD	10 (15.4)
Dementia	2 (3.1)
COPD	4 (6.1)
Peptic ulcer	2 (3.1)
Liver disease	1 (1.5)
CTD	4 (6.2)
Cancer or AIDS	9 (13.8)
Leukemia/Lymphoma	1 (1.5)
Period on PD, months	22.00 [42.00]
CAPD as dialysis modality, *n* (%)	59 (90.8)
Icodextrine solution used, *n* (%)	10 (15.4)
Daily ultrafiltration, L	1.10 [0.90]
Daily urine volume, L	0.80 [1.10]
Total Kt/V	2.45 ± 0.59
CCr, L/week	78.80 [50.38]
PET	
Glc	0.46 ± 0.15
Cr	0.65 ± 0.13
CCI	5.00 [4.00]
Composite outcome, *n* (%)	36 (55.4)
Outcome Death, *n* (%)	23 (35.4)

ESKD—end-stage kidney disease; GN—glomerulonephritis; TIN—tubulointerstitial nephritis; ADPKD—autosomal dominant polycystic kidney disease; CMP—cardiomyopathy; AMI—acute myocardial infarction; CVI—cerebrovascular insult; PVD—peripheral vascular disease; COPD—chronic obstructive pulmonary disease; CTD—connective tissue disease; PD—peritoneal dialysis; CAPD—continuous ambulatory peritoneal dialysis; Kt/V—dialysis dose index; CCr—creatinine clearance; PET—peritoneal equilibration test; Glc—glucose; Cr—creatinine; CCI—Charlson comorbidity index; AIDS—acquired immunodeficiency syndrome. Continuous variables are expressed as mean ± SD for normally distributed data and as median [interquartile range] for non-normally distributed data. Categorical variables are presented as absolute numbers (*n*) and percentages (%).

**Table 2:** Baseline laboratory values of the study population (*N* = 65).

Variables	Total
FAR	0.14 [0.04]
NLR	3.27 [2.89]
CRP, mg/mol	3.90 [6.90]
sGlc, mmol/L	5.40 [2.40]
sUr, mmol/L	15.13 ± 4.55
sCr, mmol/L	721.51 ± 207.44
Chol, mmol/L	4.73 [1.57]
TG, mmol/L	1.56 [0.88]
PTH, pg/mL	409.11 ± 248.92
Alb (g/L)	36 [28–40]
Ly (×10^9^/L)	1.6 [0.9–2.3]

FAR—fibrinogen-to-albumin ratio; NLR—neutrophil-to-lymphocyte ratio; CRP—C-reactive protein; sGlc—serum glucose; sUr—serum urea; sCr—serum creatinine; Chol—total cholesterol; TG—triglycerides; PTH—parathyroid hormone; Alb—serum albumin; Ly—lymphocytes. Continuous variables are expressed as mean ± SD for normally distributed data and as median [interquartile range] for non-normally distributed data.

**Table 3:** Comparison of baseline demographic and clinical characteristics between PD technique survival and composite outcome groups after 18 months of follow-up.

Variable	PD Technique Survival (*n* = 29)	Composite Outcome (*n* = 36)	*p*-Value
Age, years	61 [25]	65 [12]	n.s.
Sex, *n* (%) Male Female			
	15 (51.7)	20 (55.6)	n.s.
	14 (48.3)	16 (44.4)	n.s.
Icodextrine solution used, *n* (%) Yes No	4 (13.8) 25 (86.2)	30 (83.3) 6 (16.7)	n.s.
Dialysis modality, *n* (%) CAPD APD	27 (93.1) 2 (6.9)	32 (88.9) 4 (11.1)	n.s.
Period on PD, months	24.00 [35]	20.50 [53]	n.s.
Daily ultrafiltration, L	1.10 [0.75]	1.00 [1.05]	n.s.
Daily urine volume, L	1.00 [1.10]	0.60 [1.10]	n.s.
Total Kt/V	2.52 ± 0.53	2.40 ± 0.64	n.s.
CCr, L/week	92.84 ± 36.49	83.29 ± 20.02	n.s.
PET Glc	0.46 ± 0.13	0.46 ± 0.17	n.s.
PET Cr	0.66 ± 0.13	0.65 ± 0.13	n.s.
CCl	5.59 ± 2.96	6.39 ± 2.50	n.s.
Primary cause of ESKD, *n* (%) Hypertensive nephrosclerosis Diabetic nephropathy Chronic GN ADPKD TIN Other	14 (56.0) 4 (22.2) 9 (69.2) 0 (0) 0 (0) 2 (33.3)	11 (44.0) 14 (77.8) 4 (30.8) 1 (100) 2 (100) 4 (66.7)	0.036
Comorbidities, *n* (%)			
CMP No Yes	27 (50.9) 2 (16.7)	26 (49.1) 10 (83.3)	0.031
AMI No Yes	25 (47.2) 4 (33.3)	28 (52.8) 8 (66.7)	n.s.
CVI No Yes	26 (43.3) 3 (60.0)	34 (56.7) 2 (40.0)	n.s.
PVD No Yes	25 (45.5) 4 (40.0)	30 (54.5) 6 (60.0)	n.s.
Dementia No Yes	28 (44.4) 1 (50.0)	35 (55.6) 1 (50.0)	n.s.
COPD No Yes	27 (44.3) 1 (33.3)	34 (55.7) 2 (66.7)	n.s.
Peptic ulcer No Yes	29 (46.0) 0 (0)	34 (54.0) 2 (100)	n.s.
Liver disease No Yes	28 (43.8) 1 (100)	36 (56.2) 0 (0)	n.s.
CTD No Yes	26 (42.6) 3 (75.0)	35 (57.4) 1 (25.0)	n.s.
Cancer or AIDS No Yes	24 (42.9) 5 (55.6)	32 (57.1) 4 (44.4)	n.s.
Leukemia/lymphoma No Yes	29 (45.3) 0 (0)	35 (54.7) 1 (100)	n.s.

PD—peritoneal dialysis; CAPD—continuous ambulatory peritoneal dialysis; APD—automated peritoneal dialysis; Kt/V—dialysis dose index; CCr—creatinine clearance; PET—peritoneal equilibration test; Glc—glucose; Cr—creatinine; CCl—Charlson comorbidity index; ESKD—end-stage kidney disease; GN—glomerulonephritis; ADPKD—autosomal dominant polycystic kidney disease; TIN—tubulointerstitial nephritis; CMP—cardiomyopathy; AMI—acute myocardial infarction; CVI—cerebrovascular insult; PVD—peripheral vascular disease; COPD—chronic obstructive pulmonary disease; CTD—connective tissue disease; n.s.—not significant. Continuous variables are expressed as mean ± SD for normally distributed data and as median [interquartile range] for non-normally distributed data. Categorical variables are expressed as absolute numbers (*n*) and percentages (%).

**Table 4:** Comparison of baseline laboratory values between PD technique survival and composite outcome groups after 18 months of follow-up.

Variables	PD Technique Survival (*n* = 29)	Composite Outcome (*n* = 36)	*p*-Value
FAR	0.14 [0.04]	0.16 [0.04]	0.07
NLR	3.00 [3.46]	3.62 [2.77]	n.s.
CRP, mg/mol	1.90 [4.40]	6.25 [6.90]	0.01
sGlc, mmol/L	5.20 [1.50]	5.75 [3.10]	0.022
sUr, mmol/L	14.46 ± 3.99	15.67 ± 4.94	n.s.
sCr, mmol/L	705.86 ± 226.66	734.11 ± 192.91	n.s.
Chol, mmol/L	4.97 [1.45]	4.56 [1.80]	n.s.
TG, mmol/L	1.84 [1.23]	1.49 [0.84]	0.040
PTH, pg/mL	341.00 [299]	445.00 [294]	n.s.
Alb (g/L)	37 [30–40]	35 [29–40]	n.s.
Ly (×10^9^/L)	1.6 [0.9–2.3]	1.55 [0.8–2.8]	n.s.

PD—peritoneal dialysis; FAR—fibrinogen-to-albumin ratio; NLR—neutrophil-to-lymphocyte ratio; CRP—C-reactive protein; sGlc—serum glucose; sUr—serum urea; sCr—serum creatinine; Chol—total cholesterol; TG—triglycerides; PTH—parathyroid hormone; Alb—serum albumin; Ly—lymphocytes; n.s.—not significant.

**Table 5:** Predictors of the 18-month composite outcome (death or transfer to hemodialysis); logistic regression analysis.

Variables	B	SE	Wald	*p*-Value	OR (Exp B)	95% CI for OR
PTH	0.007	0.003	7.458	0.006	1.007	1.002–1.013
CRP	0.250	0.117	4.577	0.032	1.284	1.021–1.615
FAR (Z-score)	1.665	0.774	4.622	0.032	5.285	1.158–24.116
TG	−1.313	0.705	3.462	0.063	0.269	0.068–1.073
NLR	0.302	0.174	3.007	0.083	1.352	0.961–1.902
APD vs. CAPD	4.332	2.001	4.685	0.030	76.059	1.506–3841.786

PTH—parathyroid hormone; CRP—C-reactive protein; FAR—fibrinogen-to-albumin ratio; TG—triglycerides; NLR—neutrophil-to-lymphocyte ratio; APD—automated peritoneal dialysis; CAPD—continuous ambulatory peritoneal dialysis; OR—odds ratio; CI—confidence interval.

**Table 6:** Predictors of 18-month mortality, logistic regression analysis.

Variables	B	SE	Wald	*p*-Value	OR (Exp(B))	95% CI for OR
PTH	0.005	0.002	5.947	0.015	1.005	1.001–1.009
FAR (z-zone)	1.384	0.626	4.889	0.027	3.990	1.170–13.605

PTH, parathyroid hormone; FAR (Z-score), standardized fibrinogen-to-albumin ratio; OR—odds ratio; CI—confidence interval.

## Data Availability

The original contributions presented in this study are included in the article. Further inquiries can be directed to the corresponding author.

## Abbreviations

Following abbreviations were used in this manuscript:

CIn	Chronic inflammation
BMI	Body mass index
HD	Hemodialysis
ESKD	End-stage kidney disease
CRP	C-reactive protein
CVD	Cardiovascular disease
KRT	Kidney replacement therapy
PD	Peritoneal dialysis
NLR	Neutrophil-to-lymphocyte ratio
FAR	Fibrinogen-to-albumin ratio
UCCS	University Clinical Centre of Serbia
HTN	Hypertension
DM	Diabetes mellitus
PVD	Peripheral vascular disease
COPD	Chronic obstructive pulmonary disease
IHD	Ischemic heart disease
PET	Peritoneal Equilibration Test
Ccr	Total weekly creatinine clearance
sGlc	Serum glucose
sUr	Serum urea
sCr	Serum creatinine
Chol	Total serum cholesterol
TG	Triglycerides
PTH	Parathyroid hormone
CCI	Charlson Comorbidity Index
ROC	Receiver operating characteristic
AUC	Area under the curve
SD	Standard deviation
CAPD	Continuous ambulatory peritoneal dialysis
APD	Automated peritoneal dialysis
GN	Glomerulonephritis
ADPKD	Autosomal polycistic kidney disease
TIN	Tubulointerstitial nephritis
CMP	Cardiomyopathy
AMI	Acute myiocardial infarction
CVI	Cerebrovascular insult
AIDS	Acquired immune deficiency syndrome
CKD	Chronic kidney disease
GFR	Glomerular filtration rate
Kt/V	Peritoneal dialysis treatment adequacy
WBC	White blood cell count
CVE	Cardiovascular event
RKF	Residual kidney function
PLR	Platelet and lymphocyte ratio
hs	High-sensitivity
TNF-alpha	Tumor necrosis factor alpha
OR	Odds ratio
CI	Confidence interval
Alb	Albumin
Ly	Lymphocyte
Fib	Fibrinogen
IL-6	interleukin 6
CAR	C-reactive protein-to-albumin ratio
MIA	malnutrition–inflammation–atherosclerosis
